# Transient versus Permanent MCA Occlusion in Mice Genetically Modified to Have Good versus Poor Collaterals

**DOI:** 10.20900/mo.20190024

**Published:** 2019-11-27

**Authors:** Hua Zhang, James E. Faber

**Affiliations:** Department of Cell Biology and Physiology, Curriculum in Neuroscience, McAllister Heart Institute, University of North Carolina at Chapel Hill, Chapel Hill, NC 27599, USA

**Keywords:** ischemic stroke, MCA ligation, permanent, transient, collaterals

## Abstract

Collateral-dependent blood flow is capable of significantly lessening the severity of stroke. Unfortunately, collateral flow varies widely in patients for reasons that remain unclear. Studies in mice have shown that the number and diameter of cerebral collaterals vary widely due primarily to polymorphisms in genes, e.g., *Rabep2*, involved in their formation during development. However, understanding how variation in collateral abundance affects stroke progression has been hampered by lack of a method to reversibly ligate the distal middle cerebral artery (MCAO) in mice. Here we present a method and examine infarct volume 24 h after transient (tMCAO, 90 min) versus permanent occlusion (pMCAO) in mice with good versus poor collaterals. Wildtype C57BL/6 mice (have abundant collaterals) sustained small infarctions following tMCAO that increased 2.1-fold after pMCAO, reflecting significant penumbra present at 90 min. Mutant C57BL/6 mice lacking *Rabep2* (have reduced collaterals) sustained a 4-fold increase in infarct volume over WT following tMCAO and a smaller additional increase (0.4-fold) after pMCAO, reflecting reduced penumbra. Wildtype BALB/cBy (have a deficient *Rabep2* variant and poor collaterals) had large infarctions following tMCAO that increased less (0.6-fold) than the above wildtype C57BL/6 mice following pMCAO. Mutant BALB/cBy mice (have deficient *Rabep2* replaced with the C57BL/6 variant thus increased collaterals) sustained smaller infarctions after tMCAO. However, unlike C57BL/6 versus *Rabep2* mice, penumbra was not increased since infarct volume increased only 0.3-fold following pMCAO. These findings present a murine model of tMCAO and demonstrate that neuroprotective mechanisms, in addition to collaterals, also vary with genetic background and affect the evolution of stroke.

## INTRODUCTION

Reversible occlusion of the middle cerebral artery (MCA) in mice is commonly used to model thrombo-embolic stroke in humans in which the obstruction reverses spontaneously or following treatment with a thrombolytic drug or removal by thrombectomy. The most widely used method consists of inserting a silicone-coated monofilament suture into the external carotid, advancing it through the internal carotid until the tip wedges in the distal M1-MCA, and then removing it after a prescribed duration of occlusion [1–5]. While the method has the advantages of ease of performance and not requiring craniotomy, it also has several disadvantages that can be difficult to detect and mitigate. These include risk of incomplete occlusion and variable interference in flow caused by the filament itself and its promotion of thrombus formation. Both effects can obstruct flow in the lenticulo-striate arteries that branch from the proximal M1-MCA and in the arteries of the circle of Willis and their perforators near the origin of the MCA. Thrombo-emboli can also shed into the ipsilateral MCA tree and circle of Willis, the latter of which exhibits significant variation in presence and diameter of the posterior communicating arteries [6], during and after withdrawal of the filament. Thus, large infarctions occur that vary in size and involve both cortical and deep structures. Other methods have been developed that differ from the filament method and also allow use of a thrombolytic drug to simulate the clinical situation, e.g., local intravascular thrombin injection via burr-hole craniotomy or insertion of an exogenously generated thrombus via carotid catheter [1–11]. However, these methods are more difficult to perform and spontaneous thrombolysis can occur, as can variation in drug-induced thrombolysis, infarct size, and appearance of multifocal lesions. While all of the above also occur in patients, experimental designs in animal studies generally benefit from approaches that minimize variation in an endpoint(s) for a given level of treatment.

As an addition to the above models, in the present study we devised a wire-hook method for transient occlusion of the mouse M1-MCA (tMCAO) just distal to the lenticulo-striate branches at the same site that is commonly used to produce permanent cautery-induced MCA occlusion (pMCAO) and that results in infarctions confined to the neocortex [12,13]. The method is a modification of one described previously that combined transient occlusion of both the proximal MCA at or near its origin as well as the ipsilateral common carotid [14,15]. We then compared it with pMCAO to examine the evolution of stroke in mice with genetically-specified differences in number and diameter (“extent” or “abundance”) of pial collaterals. Importantly, in the present study we could not have used a model that also occludes arteries/perforators supplying subcortical regions; use of such a model would have introduced confounding factors, given that unlike the pial artery trees that supply the neocortex, intracerebral trees lack collateral interconnections.

Pial collaterals are arteriole anastomoses that cross-connect a fraction of the outermost branches of the MCA, ACA and PCA trees [16–18]. If an obstruction occurs in one of the trees, they provide retrograde perfusion that can sustain a significant ischemic penumbra and lessen the expansion of infarct core. However, the amount of collateral-dependent blood flow, as estimated by collateral score on neuroimaging, varies widely among patients even when they share the same site of occlusion, the most frequent being in the M1-MCA [1–4,18–22]. Of note, collateral status in patients with large vessel occlusion is an important determinant, together with location and time since occlusion, of early infarct volume, penumbra volume, response to thrombolytic and thrombectomy treatments, final infarct volume, and functional outcome [18–23].

While a number of hemodynamic and cell/molecular variables are anticipated to be involved in the variation in collateral flow observed in humans, investigating them is difficult, few studies are extant, and the primary contributors remain unclear. Recent animal studies have led to the suggestion that genetic differences in collateral abundance may be a major factor. Different strains of mice have been shown to exhibit a wide variation in infarct volume after permanent distal M1-MCAO that correlates closely with their large differences in collateral extent [24,25]. The latter has been linked to several naturally occurring polymorphisms in genes/genetic elements that are involved in collateral formation during development (collaterogenesis) and thus collateral abundance in the adult [26–29]. The first of these loci with the largest effect-size, the novel gene *Rabep2*, was recently identified in a C57BL/6 × BALB/cBy genetic mapping population [29]. Besides variation in abundance of anatomic collaterals, polymorphisms in genes and regulatory elements involved in other pathways undoubtedly also contribute to the variation in the complex mechanisms that determine early infarct core, penumbra loss and stroke progression. Chief among these are those that affect collateral lumen diameter, perfusion pressure, microvascular resistance, injury factors and neuronal/glial sensitivity to ischemia in the occluded region downstream of the collateral network, as well as those that affect reperfusion injury mechanisms if recanalization occurs.

Given the impact of stroke and emphasis on developing additional therapeutic interventions, studies are needed to disentangle the interactions between variation in anatomic collaterals with the above-mentioned factors. The recent availability of mice with targeted mutations of *Rabep2* [28,29] provides a way to begin this effort. Mutations of *Rabep2* do not affect pre- or postnatal development and growth of the general arterial-venous vasculature, capillary density, normal or tumor angiogenesis, or any other apparent phenotype other than collateral abundance [6,28,29]. Therefore, the aim of this study was to compare infarct volume following transient versus permanent MCA ligation, with the difference serving as an indicator of penumbra volume [5], in *Rabep2* mutant mice with good (abundant) versus poor (sparse) collaterals present on two different genetic backgrounds.

## MATERIALS AND METHODS

The following inbred strains of 3 to 7 months-old mice were studied: C57BL/6J (B6) wildtype (WT) and BALB/cByJ WT (lab colonies maintained with rejuvinators from Jackson Laboratories, Bar Harbor, ME, USA); B6 mice with targeted deletion of *Rabep2* (*Rabep2*^−/−^); BALB/cBy mice with the *Dce1* congenic allele (*Determinant of collateral extent-1*) containing the B6 variant of *Rabep2* introgressed in place of the BALB/cBy allele (denoted Cng7 in [29], abbreviated Cng-B6 herein). Construction of the above mutant strains, which are available from Jackson Laboratories, was described previously [29]. Body weights of the above strains, respectively (mean ± SE, *n*-size): 29 ± 1, 26; 28 ± 1, 21; 31 ± 1, 20; 27 ± 1, 17. All procedures were approved by the University of North Carolina’s Institutional Animal Care and Use Committee and the National Institutes of Health (NIH) Guide for the Care and Use of Laboratory Animals (IACUC# 18–123.0-A, April 2019). Approximately equal numbers of males and females were studied. We did not examine sufficient *n*-sizes to differentiate between sexes because a previous study found that pial collateral number and diameter did not differ with sex for the above strains [30]. Angiography was performed after perfusion-fixation at maximal dilation and filling of the pre-capillary vessels with viscosity-adjusted Microfil^®^ [31]. All collaterals between the ACA and MCA trees of both hemispheres were identified and their anatomic lumen diameters (maximally dilated) were measured at midpoint and averaged for each animal. Territories of the ACA, MCA and PCA trees evident on imaging of the dorsal cortex were measured as described [24].

**Figure 1.**
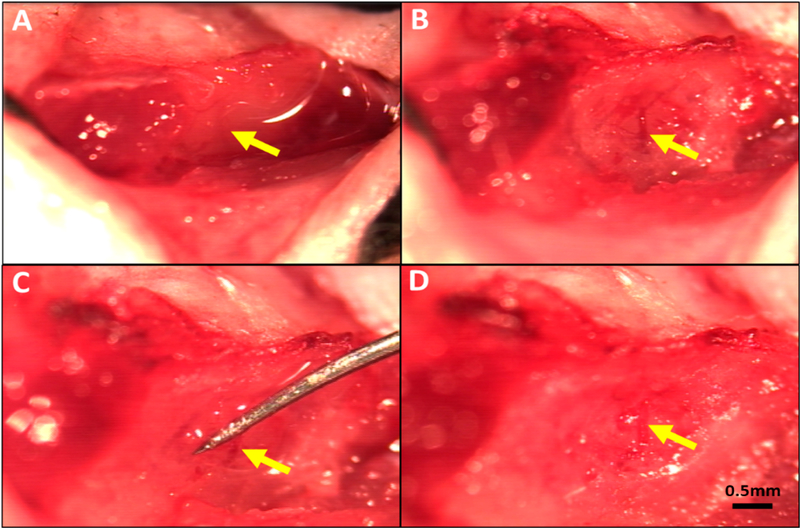
Method for reversible occlusion of the MCA distal to the lenticulostriate branches to produce transient ischemia-reperfusion in mouse. (**A**) MCA visible under the temporalis muscle. (**B**) Thinned skull window (~1 mm diameter) midway between the zygomatic arch and external auditory meatus after reflection of the temporalis muscle. (**C**) Occluder under distal M1-MCA and retracted to obstruct flow. (**D**) Flow re-established on removal of occluder after 90 min of occlusion. Magnification bar is the same for all panels.

For permanent MCA occlusion (pMCAO) [31], mice were anesthetized with ketamine and xylazine (100 and 10 mg/kg, ip) and rectal temperature was maintained at 37 ± 0.5 °C. The temporalis muscle between the right eye and ear was retracted along a 4 mm skin incision. The oblique edge of a 2.1 mm drill bit (19007–21, FST, Foster City, CA, USA) was used to thin an approximately 1 mm circle of bone overlying the distal M1-MCA. The thinned bone and dura matter were incised with a 27-gauge needle tip and reflected to expose the distal M1-MCA. The latter was cauterized (18010–00, FST, Foster City, CA, modified tip) just distal to the lenticulostriate branches. The incision was closed with suture (~15 min total surgery time), intramuscular cefazolin (50 mg/kg) and buprenorphine (0.1 mg/kg and again 12 h later) were administered, and the animal was monitored in a warmed cage during recovery from anesthesia to maintain rectal temperature. Transient MCAO (tMCAO) was performed as above with the following differences: After exposure of the distal M1-MCA at the same site as above, a wire-hook occluder fashioned from a suture needle (6147–21 5.0 MAXON CV11 taper, Covidien, eSutures.com) was positioned under the MCA and retracted sufficiently to block flow (~30 min total surgery time) (Figure 1). Light isoflurane (<1% plus 30% oxygen in room air) was then begun, and the occluder was removed 90 min after occlusion, whereupon sustained orthograde flow was confirmed. Mice were euthanized 24 h after pMCAO or tMCAO. Brains were sliced into 1 mm coronal sections that were incubated in 1% 2,3,5-triphenyltetrazolium chloride in PBS at 37 °C. Left and right forebrain hemispheres and infarcted tissue in the right hemisphere were imaged on both sides of each slice with a stereomicroscope using ImageJ software (NIH, Bethesda, MD, USA), average areas were determined for each slice, and tissue volumes were calculated. Percent infarct volume was normalized to forebrain volume [31].

Study design and statistical analysis were in accordance with the ARRIVE and STAIR guidelines [32,33]: *n*-sizes (number of animals, see figure legends) were based on our previous studies which demonstrated sufficient power to test hypotheses for the variables measured herein; Where possible the investigators were blind to mouse strain during data analysis; Individuals (replicates) of a given strain and order of strains studied were chosen at random; No data points were identified as outliers and none was excluded; The review and discussion of the literature were unbiased; Values are reported as mean ± SEM with significance defined as *p* < 0.05; Unless indicated otherwise, 1-sided *t*-tests were pre-specified according to the literature-based study-wise hypotheses that mice with greater collateral extent have larger penumbra and smaller infarct volumes following tMCAO compared to pMCAO, and that tMCAO results in larger penumbra and smaller infarct volumes compared to pMCAO for a given collateral extent.

## RESULTS

**Figure 2.**
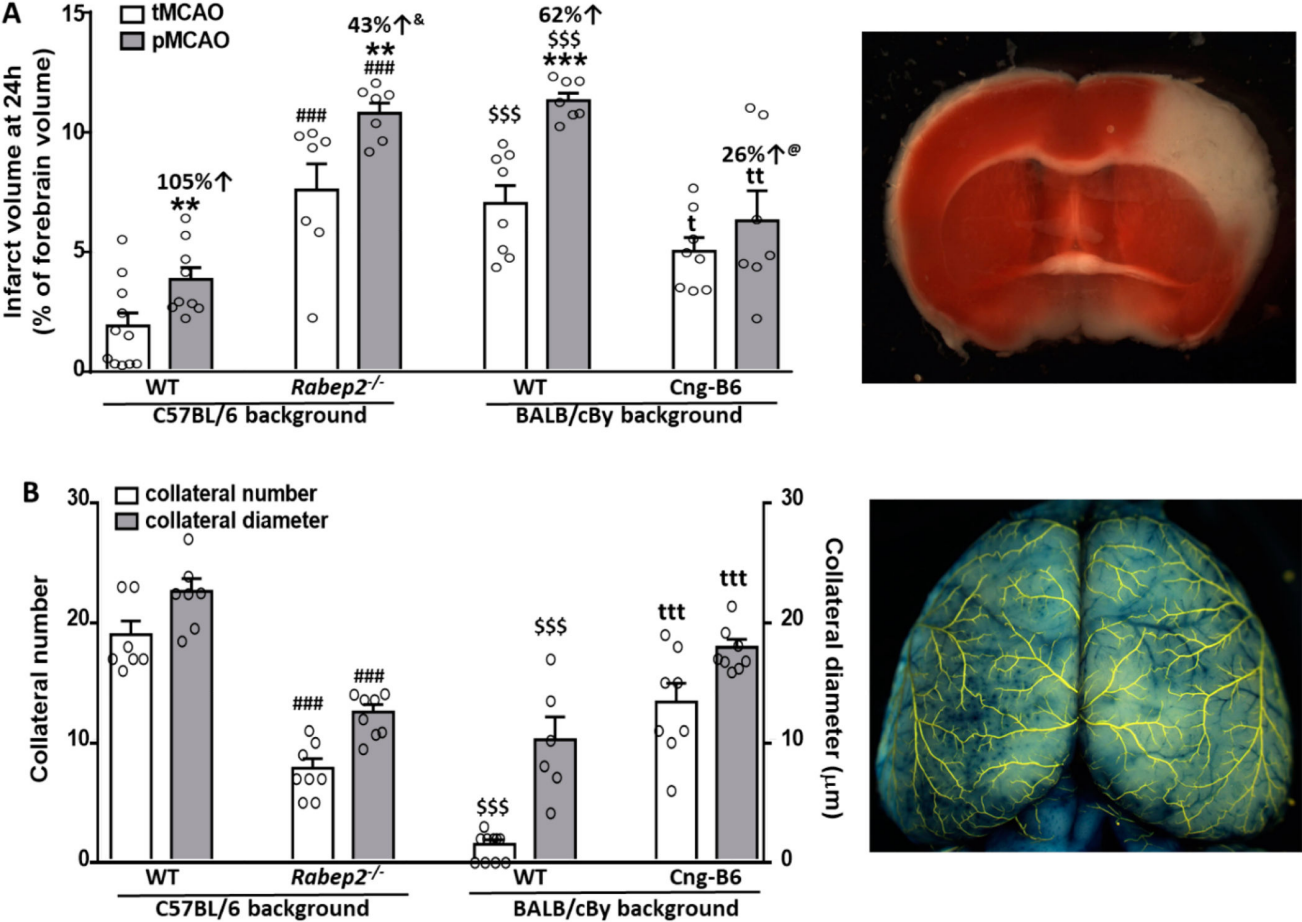
Infarct volume following transient (tMCAO, 90 min) versus permanent (pMCAO) occlusion of the distal M1-MCA in wildtype (WT) mice with abundant (C57BL/6) and sparse (BALB/cBy) collateral alleles of *Rabep2,* and mice with differences in pial collateral number and diameter produced by deletion of *Rabep2 (Rabep2*^−/−^) or by congenic introgression of the abundant-collateral B6 allele of *Rabep2* in place of the sparse-collateral BALB/cBy allele (Cng-B6). (**A**) Infarct volume at 24 h by 2,3,5-triphenyl-tetrazolium chloride. (**B**) Number and average lumen diameter of pial collaterals between the MCA and ACA trees. Number of animals for each bar, left-to-right, for (**A**): 11, 9, 7, 7, 8, 7, 8, 7; for (**B**): 7, 7, 8, 8, 10, 6 (number was zero in 4 mice), 8, 8; different animals for data in (**A**) and (**B**). Values are mean ± SEM. Pre-specified 1-sided *t*-tests: *, **, *** *p* < 0.05, 0.01, 0.001 *vs.* tMCAO; ^###^ *p* < 0.001 *vs.* C57BL/6 WT; ^t^, ^tt^, ^ttt^ *p* < 0.05, 0.01, 0.001 *vs.* BALB/cBy WT; ^$ $ $^ *p* < 0.001 BALB/cBy *vs.* C57BL/6; ^&^, *p* = 0.03 versus C57BL/6 percent change; ^@^, *p* = 0.09 versus BALB/cBy percent change.

Wildtype (WT) C57BL/6 (B6) mice had abundant large-diameter collaterals, as reported previously [7,24–26,28,29] and confirm herein, and sustained small infarctions following tMCAO that progressed to become 105% larger after pMCAO (Figure 2). This indicates a significant penumbra was present 90 min after MCAO, which is expected given their abundant collaterals. Mutant B6 mice with *Rabep2* deleted (*Rabep2*^−/−^), which have fewer collaterals with smaller diameters [29] that we confirmed herein, had 4-fold larger infarctions following tMCAO than WT mice that increased by a smaller additional amount (43%) after pMCAO, consistent with reduced penumbra as expected given their lower collateral extent.

Wildtype BALB/cBy mice, which possess a deficient *Rabep2* variant and have, compared to WT-B6 mice, a sparse number of small-diameter collaterals [7,24–26,28,29] that we confirmed herein (Figure 2), had large infarctions following tMCAO that progressed less (62% increase) than WT-B6 following pMCAO. BALB/cBy mice whose deficient *Rabep2* was replaced with the WT-B6 *Rabep2* allele by congenic introgression (Cng-B6, see Methods), have increases in collateral number and diameter when compared to wildtype BALB/cBy mice [29], findings that we confirmed herein (Figure 2). Cng-B6 mice sustained smaller infarctions after tMCAO compared to WT-BALB/cBy, as expected given their increased collateral abundance. However and unexpectedly, despite their more abundant collaterals—and unlike what was seen comparing WT-B6 versus *Rabep2*^−/−^ mice—infarct volume following pMCAO was only 26% greater (non-significant, *p* = 0.175) than that seen after tMCAO (Figure 2).

**Figure 3.**
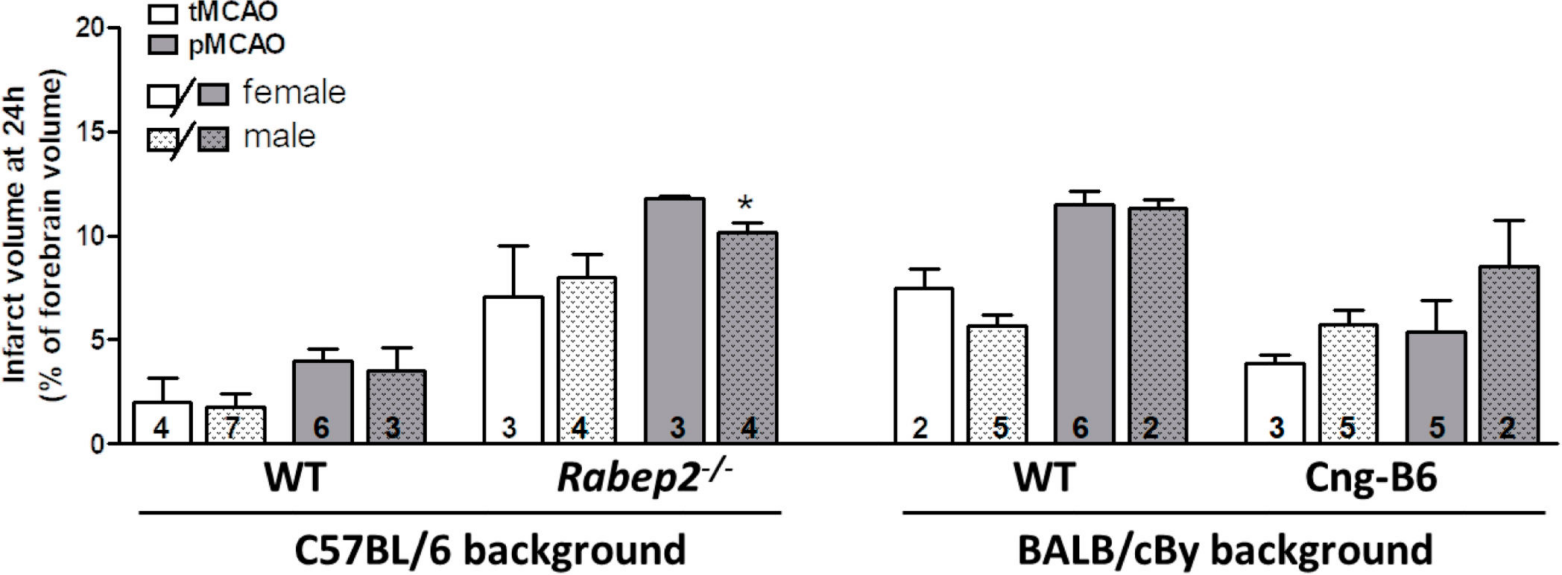
Data from Panel A of Figure 2 shown according to sex. Infarct volume following transient (tMCAO, 90 min) versus permanent (pMCAO) occlusion of the distal M1-MCA in wildtype (WT) mice with abundant (C57BL/6) and sparse (BALB/cBy) collateral alleles of *Rabep2,* and mice with differences in pial collateral number and diameter produced by deletion of *Rabep2 (Rabep2*^−/−^) or by congenic introgression of the abundant-collateral B6 allele of *Rabep2* in place of the sparse-collateral BALB/cBy allele (Cng-B6). Infarct volume at 24 h by 2,3,5-triphenyl-tetrazolium chloride. Number of animals given at base of each bar. Values are mean ± SEM. *, *p* < 0.05 versus preceding bar by 2-sided *t*-test.

The size (area/territory) of the MCA, ACA and PCA trees vary modestly in mice with differences in genetic background [24]. Since collateral number and infarct volume after pMCAO necessarily vary with the size of the MCA tree [22], we measured the territory of the MCA, ACA and PCA trees in the above strains (mean percent of total area ± SEM): C57BL/6 (*n* = 11): 51 ± 1, 36 ± 1, 13 ± 1; BALB/cBy (*n* = 10): 50 ± 1, 37 ± 2, 14 ± 1; *Rabep2*^−/−^ (*n* = 9): 53 ± 1, 35 ± 1, 13 ± 1; Cng-B6 (*n* = 9): 50 ± 1, 37 ± 1, 13 ± 1. The above values for C57BL/6 versus BALB/cBy were not significantly different, as reported previously [24], nor were values for C57BL/6 versus *Rabep2*^−/−^ or BALB/cBy versus Cng-B6. These findings are consistent with absence of a difference in body weight (see Materials and Methods), and thus predictably brain weight, for these same strain-comparisons [6]. As mentioned in Methods, we did not examine sufficient *n*-sizes to differentiate between sexes because we previously reported that pial collateral number and diameter did not differ with sex for the above strains [30]. Nevertheless, Figure 3 is provided and shows that no significant sex differences were detected (2-sided *t*-tests) except for males being slightly smaller in one comparison. However, all of the *n*-sizes were too small for reliable infarct volume comparisons, including the one that was *p* < 0.05, based on our previous studies.

## DISCUSSION

The aims of this study were two-fold: First, to devise a method for use in mice that allows transient ligation at the same site on the distal M1-MCA where permanent MCAO is commonly performed. Second, to test the hypotheses that mice with more abundant collaterals have larger penumbra and smaller infarct volumes following tMCAO compared to pMCAO, and that tMCAO results in larger penumbra and smaller infarct volumes compared to pMCAO for a given collateral extent. Our findings provide support for these hypotheses. However they also indicate that, depending on genetic background, variation in factors in addition to collaterals can augment or diminish their protective effect. Although the results are not altogether unexpected, the recent availability of mice with targeted mutations that result in differences in collateral number and diameter present on the same genetic background provided the first opportunity to test these predictions.

The level of collateral blood flow following MCA occlusion is dependent on the hemodynamic parameters specified in the Hagen-Poiseuille equation when viewed as operating in series across the ACA/PCA trees, intervening collateral network, and obstructed territory of the MCA tree. Regarding these and other determinants of collateral flow, C57BL/6 and BALB/cBy mice have large differences in collateral number and diameter (Figure 2) but no differences in collateral length, tortuosity, hematocrit (the primary determinant of blood viscosity), arterial pressure, body weight, brain weight, or average territory of their MCA, ACA and PCA trees [6,24,34,35]. Neither did arterial pressure, blood gases and pH differ after pM1-MCAO [36]. Body weight and tree territory obtained in the present study also did not differ between *Rabep2*^−/−^ and Cng-B6 mice or when compared to their above wildtype strains, in agreement with a previous study that compared C57BL/6, *Rabep2*^−/−^ and BALB/cBy [6], nor is hematocrit altered in *Rabep2*^−/−^ mice (unpublished results). These data, those in Figure 2, and the position of the collateral network as a large series-resistor between the ACA/PCA and MCA trees in the setting of MCAO, strongly support the conclusion that the genetic differences in collateral abundance in the above strains are primary determinants of their differences in infarct volume after MCAO.

Based on hemodynamic principles, collateral blood flow is in addition to the above also dependent on microvascular resistance in the obstructed tree downstream from the collaterals and changes in it that occur with time after pMCAO and following reperfusion after tMCAO. Although less significant, collateral flow can also be anticipated to be affected by changes in resistance in the trees upstream of the collateral network. A number of mechanisms are activated following MCAO that are capable of changing resistance in the MCA tree (“downstream mechanisms”), including autoregulatory vasodilation, endothelial cell activation, leukocyte and platelet activation and adhesion, hemostatic and rheologic changes, and extravascular compression from edema if present [3,17–23,37–41]. Several of these mechanisms may also occur in or affect the collateral vessels themselves, although this question has not been investigated. Unfortunately, measuring dynamic changes in the above mechanisms in the setting of abundant versus sparse anatomic collaterals during the progression of stroke following transient and permanent occlusion is difficult, resulting in a gap in our understanding.

In the present study, wildtype C57BL/6 mice with abundant collaterals sustained small infarctions after 90 min of tMCAO that doubled in size after pMCAO, whereas C57BL/6-*Rabep2* knockout mice with reduced collaterals had 4-fold larger infarctions than wildtype mice following tMCAO that increased less (43 versus 105 percent) following pMCAO (Figure 2). These results are consistent with the ability of robust collateral flow to minimize early infarct core and sustain significant penumbra tissue, and visa-versa when collateral extent is reduced. However, the absolute difference in infarct volume between pMCAO and tMCAO was not larger in *Rabep2*^−/−^ mice than WT mice despite their lower collateral extent. We speculate on two processes favored by the larger infarctions in the *Rabep2*^−/−^ mice that may contribute to this unexpected outcome: (1) A shift during MCAO in perfusion within the capillary plexus in the watershed area, which is shared anatomically by the three trees at baseline [42], due to the lower pressures in the MCA and its venous outflow pathways, resulting in retrograde perfusion of the outermost region of the MCA territory. That is, a shift in the functional watershed line between the three trees toward the MCA tree caused by “co-option” of the portion of the capillary plexus that normally receives orthograde perfusion from the MCA. And (2) diffusion of oxygen to the MCA territory from the outer territories of the ACA and PCA trees. These processes would not be active in the C57BL/6 wildtype mice (and Cng-B6, discussed below), given their abundant collaterals and small infarct cores located well away from watershed zone.

Both expected and unexpected findings were also evident for mice on the BALB/cBy background. Infarct volumes after tMCAO and pMCAO were larger in BALB/cBy wildtype mice and increased less (62 versus 105 percent) than C57BL/6 mice, as expected given their lower collateral extent. However, despite their poor collaterals, post-occlusion infarct volumes in BALB/cBy mice were not greater than *Rabep2*^−/−^ mice and their volume-difference between tMCAO and pMCAO, as an indicator of penumbra volume, was similar to C57BL/6 wildtype and *Rabep2*^−/−^ mice. One interpretation of this finding is that the BALB/cBy strain harbors a protective genetic variant(s) involved in one or more of the “downstream mechanisms” mentioned above. In support, BALB/c mice evidence less induction of inflammatory markers and neuronal cell loss during hypoxia and other types of stress, compared to C57BL/6 mice ([43,44] and references therein). Such a difference could add to the contributions of capillary co-option and oxygen diffusion described above that are favored by the larger infarctions in the *Rabep2*^−/−^ and BALB/cBy strains. Also, BALB/cBy mice average one collateral between the PCA and MCA trees (C57BL/6 average four, unpublished), which were not quantified in this study due to their out-of-focus location adjacent to the transverse sinus.

Infarct volumes after tMCAO and pMCAO were smaller in Cng-B6 mice than their BALB/cBy wildtype strain, as expected given their increased collateral extent. However, despite the much more abundant collaterals in Cng-B6, their increase in infarct volume after pMCAO versus after tMCAO was less (26%), rather than more, than that seen in BALB/cBy wildtype mice (62%). These and the other unexpected results discussed above suggest that, while the overall findings in Figure 2 indicate that genetic-dependent differences in collateral abundance are major contributors to differences in penumbra and stroke volumes, genetic variation in downstream mechanisms likely also contribute to the pace and severity of infarct progression. This conclusion is also supported by the similar infarct volumes after tMCAO and pMCAO in *Rabep2*^−/−^ and BALB/cBy wildtype mice despite the much smaller collateral number in the latter strain.

A limitation of our study is that we did not assess blood flow and other parameters (e.g., protein synthesis, energy metabolism, apoptosis) in different regions of the MCA territory after pMCAO that are required to measure penumbra volume directly [3,23,37–45]. Nor did we examine them for the potential interplay, upon reversal of occlusion, between orthograde flow and collateral-dependent retrograde flow and the reperfusion injury mechanisms that follow and increase final infarct volume. Instead, we measured the difference in 24-h infarct volume after pMCAO versus 90 min after tMCAO as a rough proxy for evolution of penumbra and core volumes [5]. Although methods required to measure the above parameters are difficult to perform in mice, such studies are needed to examine the interactions among collaterals and downstream mechanisms that govern the volume of salvageable penumbra and irreversibly injured core that both evolve during the progression from the acute to the subacute phase of stroke. Infarct volume was measured 24 h after MCAO because this time-point is commonly used in rodent studies to capture the majority of infarct progression. However, additional progression may have been evident at a later time-point. Collateral diameter averaged 23, 13, 10 and 18 microns in, respectively, C57BL6/6-WT, *Rabep2*^−/−^, BALB/cBy-WT and CngB6 mice that did not receive MCAO (Figure 2), in agreement with values previously reported for them [29]. These differences in baseline diameters could affect the percent changes in infarct volume between tMCAO and pMCAO that were determined at 24 h, since outward remodeling of collateral diameter after pMCAO may differ depending on diameter at baseline. However, a previous time-course analysis of C57BL/6 and BALB/cBy mice [24], whose baseline diameters bracket those in the *Rabep2* and CngB6 strains (Figure 2), found that maximal remodeling was achieved between 3 and 6 days after pMCAO, with curves projecting little remodeling at 24 h (18 rather than 24 h was the first time point measured).

## CONCLUSION

We present a method for reversible occlusion of the distal M1-MCA in mice at the same site where permanent ligation is commonly performed. This method has several advantages that facilitate the study of penumbra volume and core progression with and without reperfusion, as well as mechanisms of neuroprotection and candidate therapies. Our findings confirm the expected effect of variation in collateral abundance on infarct volume following transient versus permanent occlusion and, subject to the above-mentioned limitations, on relative penumbra volume as estimated by their difference. However, our results also suggest that other factors in addition to collaterals involved in the complex pathophysiology of stroke also vary with genetic background and aid or abet the neuroprotection afforded by collateral blood flow—findings that further add to the challenge of understanding and treating stroke.

## ABBREVIATIONS

ACA: anterior cerebral artery
B6: C57BL/6 mouse strain
Cng-B6: congenic-7 BALB/cBy mutant mouse strain
MCA: middle cerebral artery
PCA: posterior cerebral artery
pMCAO: permanent middle cerebral artery occlusion
*Rabep2*: RAB GTPase binding effector protein-2 gene
tMCAO: transient middle cerebral artery occlusion
WT: wildtype

## References

[1] Carmichael ST. Rodent models of focal stroke: size, mechanism, and purpose. NeuroRx. 2005 7;2(3):396–409. 16389304 10.1602/neurorx.2.3.396 PMC1144484

[2] Fluri F, Schuhmann MK, Kleinschnitz C. Animal models of ischemic stroke and their application in clinical research. Drug Des Devel Ther. 2015 7 2;9:3445–54. doi: 10.2147/DDDT.S56071 PMC4494187 26170628

[3] McCabe C, Arroja MM, Reid E, Macrae IM. Animal models of ischaemic stroke and characterisation of the ischaemic penumbra. Neuropharmacology. 2018 5 15;134(Pt B):169–77. doi: 10.1016/j.neuropharm.2017.09.022 28923277

[4] El Amki M, Clavier T, Perzo N, Bernard R, Guichet PO, Castel H. Hypothalamic, thalamic and hippocampal lesions in the mouse MCAO model: Potential involvement of deep cerebral arteries? J Neurosci Methods. 2015 10 30;254:80–5. doi: 10.1016/j.jneumeth.2015.07.008 26213218

[5] Liu F, McCullough LD. Middle cerebral artery occlusion model in rodents: methods and potential pitfalls. J Biomed Biotechnol. 2011;2011:464701. doi: 10.1155/2011/464701 21331357 PMC3035178

[6] Faber JE, Zhang H, Rzechorzek W, Dai KZ, Summers BT, Blazek C, Genetic and environmental contributions to variation in the posterior communicating collaterals of the circle of Willis. Transl Stroke Res. 2019 4;10(2):189–203. doi: 10.1007/s12975-018-0626-y 29589286

[7] Defazio RA, Levy S, Morales CL, Levy RV, Dave KR, Lin HW, A protocol for characterizing the impact of collateral flow after distal middle cerebral artery occlusion. Transl Stroke Res. 2011 3;2(1):112–27. doi: 10.1007/s12975-010-0044-2 21593993 PMC3095390

[8] Sugimori H, Yao H, Ooboshi H, Ibayashi S, Iida M. Krypton laser-induced photothrombotic distal middle cerebral artery occlusion without craniectomy in mice. Brain Res Protoc. 2004 8;13(3):189–96. doi: 10.1016/j.brainresprot.2004.06.001 15296857

[9] Orset C, Macrez R, Young AR, Panthou D, Angles-Cano E, Maubert E, Mouse model of in situ thromboembolic stroke and reperfusion. Stroke. 2007;38(10):2771–8. doi: 10.1161/STROKEAHA.107.487520 17702959

[10] Ansar S, Chatzikonstantinou E, Wistuba-Schier A, Mirau-Weber S, Fatar M, Hennerici MG, Characterization of a new model of thromboembolic stroke in C57 black/6J mice. Transl Stroke Res. 2013;5(4):526–33. doi: 10.1007/s12975-013-0315-9 24347404 PMC4092233

[11] Chen Y, Zhu W, Zhang W, Libal N, Murphy SJ, Offner H, A novel mouse model of thromboembolic stroke. J Neurosci Methods. 2015 12 30;256:203–11. doi: 10.1016/j.jneumeth.2015.09.013 26386284 PMC4651806

[12] Clayton JA, Chalothorn D, Faber JE. Vascular endothelial growth factor-A specifies formation of native collaterals and regulates collateral growth in ischemia. Circ Res. 2008;103(9):1027–36. doi: 10.1161/CIRCRESAHA.108.181115 18802023 PMC2729271

[13] Bayerl SH, Nieminen-Kelhä M, Broggini T, Vajkoczy P, Prinz V. Lateral chronic cranial window preparation enables in vivo observation following distal middle cerebral artery occlusion in mice. J Vis Exp. 2016 12 29;(118):e54701. doi: 10.3791/54701 PMC5226636 28060307

[14] Waxham MN, Grotta JC, Silva AJ, Strong R, Aronowski J. Ischemia-induced neuronal damage: a role for calcium/calmodulin-dependent protein kinase II. J Cereb Blood Flow Metab. 1996 1;16(1):1–6. doi: 10.1097/00004647-199601000-00001 8530541

[15] Zhao L, Mulligan MK, Nowak TS Jr. Substrain- and sex-dependent differences in stroke vulnerability in C57BL/6 mice. J Cereb Blood Flow Metab. 2019 3;39(3):426–38. doi: 10.1177/0271678X17746174 29260927 PMC6421252

[16] Faber JE, Chilian WM, Deindl E, van Royen N, Simons M. A brief etymology of the collateral circulation. Atherioscler Thromb Vasc Biol. 2014;34:1854–9. doi: 10.1161/ATVBAHA.114.303929 PMC4140974 25012127

[17] Nishijima Y, Akamatsu Y, Weinstein PR, Liu J. Collaterals: Implications in cerebral ischemic diseases and therapeutic interventions. Brain Res. 2015;1623:18–29. doi: 10.1016/j.brainres.2015.03.006 25770816 PMC4567541

[18] Bang OY, Goyal M, Liebeskind DS. Collateral circulation in ischemic stroke: Assessment tools and therapeutic strategies. Stroke. 2015;46:3302–9. doi: 10.1161/STROKEAHA.115.010508 26451027 PMC4624512

[19] Ginsberg MD. The cerebral collateral circulation: Relevance to pathophysiology and treatment of stroke. Neuropharmacology. 2018;134: 280–92. doi: 10.1016/j.neuropharm.2017.08.003 28801174

[20] Rocha M, Jovin TG. Fast versus slow progressors of infarct growth in large vessel occlusion stroke: clinical and research implications. Stroke. 2017 9;48(9):2621–7. doi: 10.1161/STROKEAHA.117.017673 28794271

[21] El Amki M, Wegener S. Improving cerebral blood flow after arterial recanalization: A novel therapeutic strategy in stroke. Int J Mol Sci. 2017 12 9;18(12):E2669. doi: 10.3390/ijms18122669 PMC5751271 29232823

[22] Yu I, Bang OY, Chung JW, Kim YC, Choi EH, Seo WK, ; Calgary-Samsung Stroke Collaborators. Admission diffusion-weighted imaging lesion volume in patients with large vessel occlusion stroke and Alberta stroke program early CT score of ≥6 Points: Serial computed tomography-magnetic resonance imaging collateral measurements. Stroke. 2019 11;50(11):3115–20. doi: 10.1161/STROKEAHA.119.026229 31554502

[23] Donnan GA, Baron J-C, Davis SM, Sharp FR, editors. The ischemic penumbra. New York (US): Informa Healthcare USA; 2007.

[24] Zhang H, Prabhakar P, Sealock RW, Faber JE. Wide genetic variation in the native pial collateral circulation is a major determinant of variation in severity of stroke. J Cerebral Blood Flow Metab. 2010;30:923–34. doi: 10.1038/jcbfm.2010.10 PMC2949178 20125182

[25] Wang S, Zhang H, Wiltshire T, Sealock R, Faber JE. Genetic dissection of the Canq1 locus governing variation in extent of the collateral circulation. PLoS One. 2012;7:e31910. doi: 10.1371/journal.pone.0031910 PMC3295810 22412848

[26] Chalothorn D, Faber JE. Formation and maturation or the murine native cerebral collateral circulation. J Mol Cell Cardiol. 2010 8;49(2):251–9. doi: 10.1016/j.yjmcc.2010.03.014 20346953 PMC2885464

[27] Lucitti JL, Mackey J, Morrison J, Haigh J, Adams R, Faber JE. Formation of the collateral circulation is regulated by vascular endothelial growth factor-A and A Disintegrin and Metalloprotease Family Members 10 and 17. Circ Res. 2012 12 7;111(12):1539–50. doi: 10.1161/CIRCRESAHA.112.279109 22965144 PMC3518639

[28] Sealock R, Zhang H, Lucitti JL, Moore SM, Faber JE. Congenic fine-mapping identifies a major causal locus for variation in the native collateral circulation and ischemic injury in brain and lower extremity. Circ Res. 2014 2 14;114(4):660–71. doi: 10.1161/CIRCRESAHA.114.302931 24300334 PMC3966023

[29] Lucitti JL, Sealock R, Buckley BK, Zhang H, Xiao L, Dudley AC, Variants of Rab GTPase-effector binding protein-2 cause variation in the collateral circulation and severity of stroke. Stroke. 2016;47:3022–31. doi: 10.1161/STROKEAHA.116.014160 27811335 PMC5134893

[30] Faber JE, Moore SM, Lucitti JL, Aghajanian A, Zhang H. Sex differences in the cerebral collateral circulation. Transl Stroke Res. 2016;8:273–83. doi: 10.1007/s12975-016-0508-0 27844273 PMC5429998

[31] Zhang H, Jin B, Faber JE. Mouse models of Alzheimer’s disease cause loss of pial collaterals and increased severity of ischemic stroke. Angiogenesis. 2018;22:263–79. doi: 10.1007/s10456-018-9655-0 30519973 PMC6475514

[32] Kilkenny C, Browne WJ, Cuthill IC, Emerson M, Altman DG. Improving Bioscience Research Reporting: The ARRIVE Guidelines for Reporting Animal Research. PLoS Biol. 2010;8(6):e1000412. doi: 10.1371/journal.pbio.1000412 PMC2893951 20613859

[33] RIGOR. Improving the quality of NINDS-supported preclinical and clinical research through rigorous study design and transparent reporting. Bethesda (US): NINDS; 2012 Available from: http://www.ninds.nih.gov/funding/transparency_in_reporting_guidance.pdf Accessed 2019 Oct 4.

[34] Peng H, Yang XP, Carretero OA, Nakagawa P, D’Ambrosio M, Leung P, Angiotensin II-induced dilated cardiomyopathy in Balb/c but not C57BL/6J mice. Exp Physiol. 2011;96(8):756–64. doi: 10.1113/expphysiol.2011.057612 21602297 PMC3256574

[35] Ryan MJ, Didion SP, Davis DR, Faraci FM, Sigmund CD. Endothelial dysfunction and blood pressure variability in selected inbred mouse strains. Arterioscler Thromb Vasc Biol. 2002 1;22(1):42–8. doi: 10.1161/hq0102.101098 11788459

[36] Majid A, He YY, Gidday JM, Kaplan SS, Gonzales ER, Park TS, Differences in vulnerability to permanent focal cerebral ischemia among 3 common mouse strains. Stroke. 2000 11;31(11):2707–14. 11062298 10.1161/01.str.31.11.2707

[37] Hu X, De Silva TM, Chen J, Faraci FM. Cerebral vascular disease and neurovascular injury in ischemic stroke. Circ Res. 2017;120(3):449–71. doi: 10.1161/CIRCRESAHA.116.308427 28154097 PMC5313039

[38] Nagy Z, Nardai S. Cerebral ischemia/repefusion injury: From bench space to bedside. Brain Res Bull. 2017 9;134:30–7. doi: 10.1016/j.brainresbull.2017.06.011 28625785

[39] Venkat P, Chopp M, Chen J. Blood-brain barrier disruption, vascular impairment, and ischemia/reperfusion damage in diabetic stroke. J Am Heart Assoc. 2017 6 1;6(6):e005819. doi: 10.1161/JAHA.117.005819 PMC5669184 28572280

[40] Pham M, Bendszus M. Facing time in ischemic stroke: An alternative hypothesis for collateral failure. Clin Neuroradiol. 2016 6;26(2):141–51. doi: 10.1007/s00062-016-0507-2 26952017 PMC4914521

[41] Winship IR. Cerebral collaterals and collateral therapeutics for acute ischemic stroke. Microcirculation. 2015 4;22(3):228–36. doi: 10.1111/micc.12177 25351102

[42] Loftus CM, Greene GM, Detwiler KN, Baumbach GL, Heistad DD. Studies of collateral perfusion to canine middle cerebral artery territory. Am J Physiol. 1990 8;259(2 Pt 2):H560–6. 2386228 10.1152/ajpheart.1990.259.2.H560

[43] Walsh N, Bravo-Nuevo A, Geller S, Stone J. Resistance of photoreceptors in the C57BL/6-c2J, C57BL/6J, and BALB/cJ mouse strains to oxygen stress: evidence of an oxygen phenotype. Curr Eye Res. 2004 12;29(6):441–7. 15764088 10.1080/02713680490522416

[44] Rocha-Ferreira E, Phillips E, Francesch-Domenech E, Thei L, Peebles DM, Raivich G, The role of different strain backgrounds in bacterial endotoxin-mediated sensitization to neonatal hypoxic-ischemic brain damage. Neuroscience. 2015 12 17;311:292–307. doi: 10.1016/j.neuroscience.2015.10.035 26515746 PMC4675086

[45] Beretta S, Cuccione E, Versace A, Carone D, Riva M, Padovano G, Cerebral collateral flow defines topography and evolution of molecular penumbra in experimental ischemic stroke. Neurobiol Dis. 2015 2;74:305–13. doi: 10.1016/j.nbd.2014.11.019 25484287

